# Workforce participation among international medical graduates in the National Health Service of England: a retrospective longitudinal study

**DOI:** 10.1186/1478-4491-6-9

**Published:** 2008-05-30

**Authors:** Mark Hann, Bonnie Sibbald, Ruth Young

**Affiliations:** 1 National Primary Care Research and Development Centre, 5th Floor – Williamson Building, University of Manchester, Oxford Road, Manchester, M13 9PL, UK; 2 School of Nursing and Midwifery, Kings College London, 5th Floor – Waterloo Bridge Wing, Franklin Wilkins Building, 150 Stamford Street, London, SE1 9NH, UK

## Abstract

**Background:**

Balancing medical workforce supply with demand requires good information about factors affecting retention. Overseas qualified doctors comprise 30% of the National Health Service (NHS) workforce in England yet little is known about the impact of country of qualification on length of stay. We aimed to address this need.

**Methods:**

Using NHS annual census data, we calculated the duration of 'episodes of work' for doctors entering the workforce between 1992 and 2003. Survival analysis was used to examine variations in retention by country of qualification. The extent to which differences in retention could be explained by differences in doctors' age, sex and medical specialty was examined by logistic regression.

**Results:**

Countries supplying doctors to the NHS could be divided into those with better or worse long-term retention than domestically trained doctors. Countries in the former category were generally located in the Middle East, non-European Economic Area Europe, Northern Africa and Asia, and tended to be poorer with fewer doctors per head of population, but stronger economic growth. A doctor's age and medical specialty, but not sex, influenced patterns of retention.

**Conclusion:**

Adjusting workforce participation by country of qualification can improve estimates of the number of medical school places needed to balance supply with demand. Developing countries undergoing strong economic growth are likely to be the most important suppliers of long stay medical migrants.

## Background

The National Health Service (NHS) of England depends heavily on the services of doctors who obtained their primary medical qualification outside the country. International medical graduates constitute 32% of the medical workforce with more than one-in-three hospital doctors and one-in-five general practitioners having qualified overseas. To reduce this dependency and sustain overall workforce growth, five new medical schools were introduced in 2000, increasing graduate output by 60% [1]. Anecdotal evidence suggests that, by 2006, domestic supply may have exceeded demand with new graduates finding it difficult to obtain employment. The government accordingly withdrew permit-free immigration for doctors in April 2006 and NHS employers must now appoint doctors who qualified within the European Union in preference to others [2]. The negative impact this has had on international medical graduates, following as it does on a period of active recruitment abroad, may have a lasting effect on England's ability to compete successfully in the global market for medical labour [1,3].

Avoiding future imbalance between supply and demand is not easy. Effective workforce planning requires, among other things, good information about workforce participation patterns among doctors. Projected demand for doctors can then be translated into the number of medical school places needed to meet that demand. Estimating the extent to which medical school output in England may need adjustment is made difficult by the paucity of information about retention rates among international medical graduates. We aimed to address this information need. NHS annual census data from 1991 to 2004 were used to investigate variations in the length of stay of doctors by their country of first qualification.

## Methods

England conducts annual censuses of all doctors in contract to the NHS. Using Stata [4], annual datasets for 1991 to 2004 were linked chronologically by matching doctors' unique General Medical Council registration number to construct a retrospective time series of doctors' participation in the NHS workforce. The data contained work records for 200 046 individual doctors.

### Workforce Participation

The data allow us to determine whether a doctor entered, remained in, or exited the workforce between consecutive census dates. A doctor was deemed to have entered the workforce if they appeared in the dataset in a particular year having been absent the previous year. A doctor was deemed to have exited the workforce if they no longer appeared in the dataset in a particular year, having been present the previous year. The duration of an episode of work was then calculated as the number of consecutive years between entry and exit. The true duration of an episode of work may vary by up to one year above or below this estimate, but cannot be determined with greater precision from the available data. Over the course of their career, doctors may have many episodes of work separated by short or long breaks. Our analysis focused on doctors' first observed episodes of work. Analyses not reported here showed that subsequent work episodes, where determinable, were no shorter in duration and did not differ from first episodes in their rank association with doctors' country of qualification. Total years in the workforce cannot be estimated as we cannot know whether the final observed exit from the dataset is permanent.

The analysis was restricted to the 92 679 doctors with full registration who began an episode of work between 1992 and 2003 and whose country of qualification was known. The 70 906 doctors with full registration who were already in the workforce in 1991 were excluded as their year of entry was unknown. The 22 706 doctors with limited registration (which precedes full registration) were also excluded as there is no link between limited and full registration identifiers.

### Analyses

Variation in the duration of first episodes of work by country of qualification was examined in two ways. First doctors who qualified outside the UK were grouped by geographic region as follows (see Appendix for regional definitions): European Economic Area (EEA); non-EEA Europe; South Asia; Australasia; Americas; Middle East; Northern Africa; and Sub-Saharan Africa. Differences across regions were then examined. Next we selected the 20 countries supplying the highest numbers of doctors and examined differences across countries.

Data were analysed using survival analysis [5]. This measures the span of time ending in an event, where the 'event' for the purpose of this study is exit from the workforce. A defining feature of survival data is that the event may not be observed for some individuals. In the context of our data, this means we cannot determine the duration of an episode of work that continues beyond the end of our observation period. Survival analysis takes this uncertainty into account. Kaplan-Meier estimates of the proportion of doctors remaining in the workforce by number of years since entry were plotted by geographical region and for the 20 leading supply countries. As there was no wider reference population to which we could generalize, formal hypothesis tests of differences between groups were not conducted.

We then used logistic regression to model the effects of age, sex and medical specialty (general practice vs. hospital) on survival in the workforce for at least 3 years after entry as this was the median length of stay across all doctors. Sensitivity analyses considered shorter (2+ years) and longer (4+ years) lengths of stay. In these models, the linear and quadratic effects of age, and the binary variables denoting sex and medical specialty were all interacted with geographical region: as such, their main effects were construed from visual analysis of survival graphs. We did not use Cox's proportional hazards regression analysis because our data did not meet the proportionality assumption. We also considered and rejected discrete-time survival analysis due to the obvious co-linearity between time and age.

## Results

### Variation by Geographic Region

**Figure 1:**
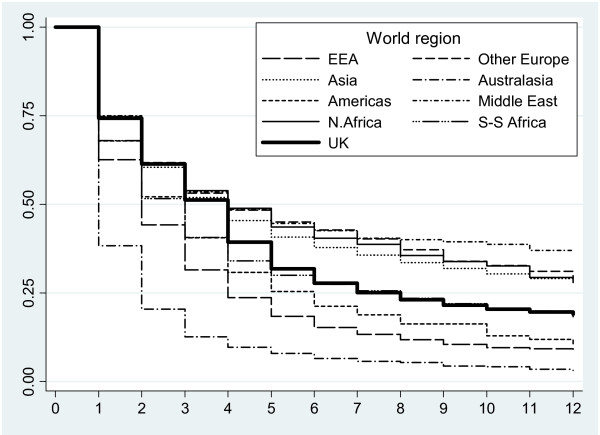
Proportion of doctors remaining in the workforce by number of years since entry.

Table 1 shows the numbers of doctors entering the workforce and the median duration of their first episodes of work for each geographical region. Of 92,679 doctors entering the workforce between 1992 and 2003, 39,061 (42%) had qualified outside the UK. Figure 1 shows how the percentage of doctors retained in the workforce declines with time for each geographical region. Doctors who qualified in the Middle East, non-EEA Europe, Northern Africa and Asia were alike in having similar first lengths of stay (median 4 years) to UK qualified doctors. While short-term retention in the workforce was similar to that of UK qualified doctors, longer term retention tended to be better. In contrast, doctors who qualified in Australasia, the EEA, the Americas and Sub-Saharan Africa had shorter first episodes of work (median 1 – 3 years) than UK qualified doctors. All except Sub-Saharan Africa had lower rates of retention in both the short and long term. Sub-Saharan Africa had worse short-term retention but similar long-term retention to the UK.

**Table 1:** Number and duration of doctors' first episodes of work in the NHS by country of qualification region

Country of qualification region	First episodes of work		Duration of first episode [Median (IQR)]
		
	Total	Average annual	
United Kingdom	53 618	4468	4 (1, 8)
Asia	16 564	1380	4 (1, .)
European Economic Area	8 969	747	2 (1, 4)
Sub-Saharan Africa	5042	420	3 (1, 8)
Australasia	2747	229	1 (1, 2)
Northern Africa	1741	145	4 (1, .)
Middle East	1666	139	4 (1, .)
Other European (non-EEA)	1299	108	4 (1, .)
Americas	1033	86	3 (1, 6)

ALL REGIONS	92 679	7,723	3 (1, 7)

Note: IQR = inter-quartile range; . = upper quartile could not be calculated (fewer than 75% leave the workforce)

Table 2 shows the differences between geographic regions in doctors' age, sex and medical speciality. It is evident the majority of doctors enter hospital specialties. Those who qualified in the EEA most closely resemble UK qualified doctors in terms of their age and sex; while those who qualified elsewhere are more frequently male and/or older. The reasons for this are unclear. Different countries have different training regimes: the UK, for example, is five years undergraduate, whereas the USA is four years postgraduate. However, this is unlikely to completely explain such variation. The most likely explanation is that migrants are simply older than domestically trained doctors. They may choose to gain experience in their own country for a number of years, before moving to the UK, or perhaps their decisions are economically or politically driven.

**Table 2:** Doctors' age, sex and medical speciality on entry to the NHS by country of qualification region

Country of qualification region	Sex N (%)		Age in years N (%)					Specialty N (%)	
	
	M	F	Average	<30	30 – 39	40 – 49	50 – 59	Gen. Prtc.	Hospital
United Kingdom	27 877 (52.0)	25 739 (48.0)	27.7	40 941 (77.2)	8361 (15.8)	2256 (4.2)	1505 (2.8)	3434 (6.4)	50 184 (93.6)
European Economic Area	5267 (58.7)	3701 (41.3)	32.4	2955 (33.6)	4999 (56.8)	632 (7.2)	212 (2.4)	867 (9.7)	8102 (90.3)
Australasia	1793 (65.3)	954 (34.7)	32.4	955 (35.1)	1526 (56.2)	160 (5.9)	66 (2.8)	118 (4.3)	2629 (95.7)
Americas	619 (59.9)	414 (40.1)	33.3	370 (35.9)	488 (47.3)	131 (12.7)	42 (4.1)	12 (1.2)	1021 (98.8)
Sub-Saharan Africa	3474 (68.9)	1568 (31.1)	34.0	1555 (31.0)	2521 (50.3)	757 (15.1)	182 (3.6)	219 (4.3)	4822 (95.7)
Asia	11 885 (71.8)	4679 (28.2)	35.5	3193 (19.3)	9623 (58.3)	2705 (16.4)	982 (6.0)	688 (4.2)	15 876 (95.8)
Other European (non-EEA)	756 (58.2)	543 (41.8)	36.4	218 (16.8)	732 (56.6)	255 (19.7)	89 (6.9)	60 (4.6)	1239 (95.4)
Middle East	1299 (78.0)	367 (22.0)	37.5	165 (9.9)	944 (56.9)	449 (27.1)	102 (6.1)	78 (4.7)	1588 (95.3)
Northern Africa	1534 (88.1)	207 (11.9)	38.7	63 (3.6)	992 (57.2)	592 (34.1)	88 (5.1)	54 (3.1)	1687 (96.9)

ALL REGIONS	54 504 (58.8)	38 172 (41.2)	30.6	50 415 (54.9)	30 186 (32.9)	7937 (8.6)	3278 (3.6)	5530 (6.0)	87 148 (94.0)

Note: Gen. Prtc. = general practice-based

**Figure 2:**
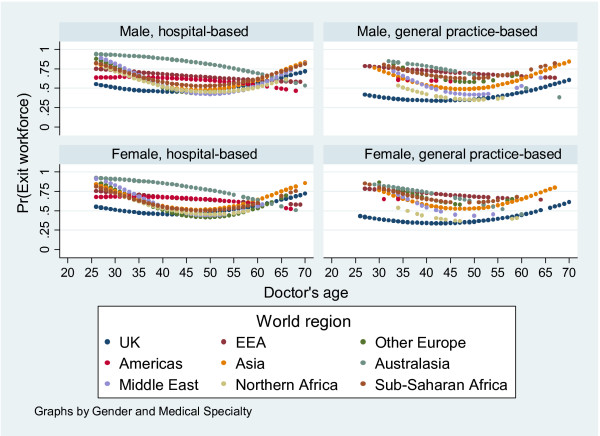
Predicted probability of a doctor leaving the workforce before or during their third year by age, sex and medical specialty.

Figure 2 shows how the predicted probability of remaining in the workforce for at least 3 years is affected by doctors' age, sex and specialty. Sex has no apparent effect on the probability of leaving the workforce; but age and medical specialty do. Among UK-qualified doctors, the probability of leaving the workforce declines up to age 40–45 years (the 'turning point'), then increases. This pattern is also observed for non-EEA European, Asian, Middle Eastern and African qualified doctors, although the turning point shifts to 45–50 years. In contrast, for EEA and Australasian qualified doctors, the probability of leaving declines continuously. For American-qualified doctors there is actually a slight increase up to 35 years, followed by a steady decline. Doctors in general practice (GPs) are less likely to leave than hospital doctors, but this effect is primarily attributable to UK qualified GPs. There is, for example, no obvious difference between Asian qualified GPs and hospital doctors. The effects of age, sex and specialty on the probability of leaving the workforce within 2 or 4 years after entry were the same as for 3 years (data not shown); the results are therefore robust.

### Variation by country

Table 3 shows the numbers of doctors entering the workforce and the median duration of first episodes of work for each of the 20 leading countries supplying doctors to England. Countries are additionally profiled in terms of their Gross Domestic Product (GDP) per capita, real growth rate in GDP, and numbers of doctors per 1000 population. The leading supplier country was India followed by Germany, Pakistan, South Africa, Australia, Republic of Ireland, Nigeria, Egypt, Greece, Iraq, Spain, Sri Lanka, Italy, Jamaica, New Zealand, Netherlands, Belgium, Sudan, Libya and Myanmar. Of the 39 061 medical migrants entering the NHS between 1992 and 2003, 34 778 (89%) qualified in one of these 20 countries.

**Table 3:** Top 20 Countries Supplying Doctors to the NHS 1992 – 2003

Country of qualification	First episodes of work		Duration of first episode [median (IQR)]	Gross domestic product (GDP)		Doctors per 1000 Population
				
	Total	Average Annual		GDP per capita ($)	Real growth rate (%)	
Suppliers of long term migrants						

India	12 381	1032	4 (2, .)	3300	7.6	0.60_2005_
Pakistan	2402	200	5 (2, .)	2400	6.9	0.74_2004_
Nigeria	1585	132	3 (1, 11)	1400	6.2	0.28_2003_
Egypt	1336	111	4 (1, .)	3900	4.9	0.54_2003_
Iraq	983	82	5 (2, .)	3400	-3.0	0.66_2004_
Sri Lanka	763	64	3 (1, .)	4300	5.6	0.55_2004_
Sudan	429	36	4 (1, 10)	2100	7.0	0.22_2004_
Libya	389	32	4 (2, 11)	11 400	8.5	1.29_1997_
Myanmar	375	31	4 (1, .)	1700	2.9	0.36_2004_

mean				3767	5.2	0.58

Suppliers of Short Term Migrants						

Germany	2673	223	2 (1, 4)	30 400	0.9	3.37_2003_
South Africa	2386	199	2 (1, 5)	12 000	4.9	0.77_2004_
Australia	2137	178	1 (1, 2)	31 900	2.5	2.47_2001_
Rep. Ireland	1982	165	3 (1, 7)	41 000	4.7	2.79_2004_
Greece	1057	88	2 (1, 3)	22 200	3.7	4.38_2001_
Spain	949	79	3 (1, 5)	22 500	3.4	3.30_2003_
Italy	652	54	2 (1, 4)	29 200	0.1	4.20_2004_
Jamaica	610	51	3 (1, 5)	4400	1.5	0.85_2003_
N. Zealand	610	51	1 (1, 3)	25 200	2.2	2.37_2001_
Netherlands	600	50	2 (1, 3)	30 500	1.1	3.15_2003_
Belgium	479	40	1 (1, 2)	31 400	1.5	4.49_2002_

mean				25 518	2.4	2.92

Notes

IQR: inter-quartile range; . = upper quartile could not be estimated (fewer than 75% leave the workforce).

GDP: all figures are 2005 estimates (data from the CIA World FactBook [6]).

Doctors per 1000 Population: subscript represents 'year of estimate' (data from the 2006 World Health Report, conducted by the WHO [7]).

Kaplan-Meier estimates (not presented here) indicate that these 20 countries form two distinct groups with very different patterns of workforce participation: better long term retention than the UK or worse retention than the UK. Countries with better long term retention than the UK include those in Asia (India, Pakistan, Sri Lanka, and Myanmar), the Middle East (Iraq) and Northern Africa (Egypt and Libya) together with the Sub-Saharan countries of Nigeria and Sudan. Of all medical migrants, 20 643 (53%) were from these countries. Countries with worse retention than the UK include those in Australasia (Australia and New Zealand) and the EEA (Belgium, Germany, Greece, Italy, Netherlands, Spain and Republic of Ireland), together with Jamaica and South Africa. Of all medical migrants, 36% (14 135) were from these countries.

Table 3 also shows that, as compared with countries supplying short term migrants, those supplying long term migrants tend to be poorer (average GDP per capita of US$ 3767 versus US$ 25 518) with a lower number of doctors per 1000 population (mean of 0.58 versus 2.92) but stronger economic growth (mean GDP real growth rate of 5.2% versus 2.4%) [6,7].

There are exceptions to this profile. Among countries supplying long term migrants to the NHS, Iraq and Myanmar have lower than expected economic growth rates. Among countries supplying short term migrants to the NHS, South Africa and Jamaica have a lower than expected GDP per capita.

## Discussion

Forty-two percent of doctors entering the NHS between 1992 and 2003 obtained their primary medical qualification outside the UK and, of these, 89% were from one of 20 countries. Among these 20 leading supply countries, two broad patterns of workforce participation were discerned. Eleven countries (providing 36% of all migrants) supplied 'short-term migrants' characterized by short first episodes of work (median 1 – 3 years) and low rates of retention in both the short and longer term. Nine countries (providing 53% of all migrants) supplied 'long-term migrants' characterized by longer first episodes of work (median 3 – 5 years) with similar rates of retention to domestically trained doctors in the short term and better rates of retention in the longer term. The same broad patterns were discernable in subsequent episodes of work beyond the first, suggesting that first episodes of work are a marker for lifetime service in the NHS although longer term studies than ours would be needed to confirm this.

Differences in retention among doctors from different countries were associated with differences in doctors' age and medical specialty. Retention in the workforce was generally higher for middle-aged doctors and those working in general practice as opposed to hospital medicine. The age effect is unsurprising; younger doctors are more likely to have career breaks while older doctors are more likely to retire. UK qualified GPs are less likely to leave because the majority own the organizations in which they work and this constrains mobility. Sex had no discernable effect on retention which is surprising given that women take career breaks to bear children. As our data show that men are as likely as women to leave the workforce, their reasons for so doing merit further study.

The findings need to be considered within the overall limitations of the study. We excluded doctors already in the workforce in 1991 and those with limited registration for whom length of stay could not be measured. This unavoidable truncation of the data means we will have underestimated actual lengths of stay but should not affect observed differences between countries. In addition we have no information about doctors employed entirely outside the NHS; although these are thought to form a very small percentage of the workforce.

Previous research suggests that economic factors are likely to be among the most important drivers of international medical migration [8-10]. All other factors being equal, workers tend to move to areas that maximise the return on their labour. Disparities in economic opportunity between countries thus define a 'push-pull' gradient that creates the impetus for international labour movement. Our findings support this view in showing that short term medical migrants to England tend to be from wealthy, socially stable countries with high numbers of doctors per head of population. In contrast, long-term migrants to England tend to come from developing countries with few doctors per head of population but rapidly growing economies. Such countries may have the wealth to train doctors but not the resources to meet their career and lifestyle expectations [8-10].

There are, however, exceptions to this profile. Iraq, Myanmar and Jamaica resemble each other in being poor countries with weak economic growth rates and few doctors per head of population. They are also distinguished by socio-political instability which has been shown elsewhere to drive medical emigration [11]. South Africa more closely resembles countries supplying long term migrants in terms of its economic profile; although here too socio-political instability is a push factor to emigration [12]. It is therefore understandable that Iraq and Myanmar supply long term migrants to England but unclear why South Africa and Jamaica supply short term migrants. It may be that doctors from these latter two countries are seeking long term residence abroad and use England as a stepping stone to other developed countries.

## Conclusion

While we can only speculate as to the factors underlying the different patterns of workforce participation, the implications for medical workforce planning in England are clear. The higher the proportion of international medical graduates from countries supplying long-term migrants, the lower will be England's demand for domestically trained doctors. Adjusting workforce participation by country of qualification should give improved estimates of the number of medical school places that will be needed to balance domestic supply with demand. The same is likely to be true for the United States, Canada and Australia whose position in the international medical labour market is similar to that of the UK [13]. Further research into the factors underpinning medical migration should lead to improved forecasting of future international flows that would also enhance workforce planning.

## Competing interests

The authors declare that they have no competing interests.

## Authors' contributions

BS and RY were instrumental in the conception and design of this project. MH constructed and analysed the database with significant input from BS. MH and BS drafted the article, which was revised following comments from RY. All authors approved the final article for publication.

## Supplementary Material

Additional file 1 **'Geographical region definitions'**. Definitions as used in this article. Click here for file
