# Supplementary material for: Workforce participation among international medical graduates in the National Health Service of England: a retrospective longitudinal study
Source: Hum Resour Health. 2008 May 30;6:9. doi: 10.1186/1478-4491-6-9 (PMC2432073; doi:10.1186/1478-4491-6-9)
Supplement: Additional file 1 — 'Geographical region definitions'. Definitions as used in this article. [file 1478-4491-6-9-S1.doc]

EEA as defined in 1994: Austria, Belgium, Denmark, Finland, France, Germany, Greece, Iceland, Rep. of Ireland, Italy, Luxembourg, Netherlands, Norway, Portugal, Spain, Sweden (& UK)

Non-EEA European countries: Albania, Bulgaria, the former Czechoslovakia, Hungary, Poland, Romania, the former Soviet Union, all former Yugoslav republics & Malta

Northern Africa defined as Algeria, Egypt, Libya, Morocco and Tunisia

Sub-Saharan Africa defined as all African countries apart from those in Northern Africa
